# Mitigating bias in radiology: The promise of topological data analysis and simplicial complexes

**DOI:** 10.18632/oncotarget.28668

**Published:** 2024-11-12

**Authors:** Yashbir Singh, Colleen Farrelly, Quincy A. Hathaway, Gunnar Carlsson

**Keywords:** topological data analysis, simplicial complexes, radiology, medical imaging

## Abstract

Topological Data Analysis (TDA) and simplicial complexes offer a novel approach to address biases in AI-assisted radiology. By capturing complex structures, n-way interactions, and geometric relationships in medical images, TDA enhances feature extraction, improves representation robustness, and increases interpretability. This mathematical framework has the potential to significantly improve the accuracy and fairness of radiological assessments, paving the way for more equitable patient care.

## INTRODUCTION

In recent years, the radiology has witnessed a rapid integration of artificial intelligence (AI) and machine learning techniques to assist in image interpretation and diagnosis [1]. While these advancements have shown great promise, they have also brought to light concerns about potential biases in AI algorithms and their impact on patient care [2]. As we strive to improve the accuracy and fairness of radiological assessments, a novel approach is emerging that holds significant potential: Topological Data Analysis (TDA) and the use of simplicial complexes.

### Introducing TDA in radiology

TDA is a mathematical framework that allows us to study the shape and structure of data [3]. In the context of radiology, TDA offers a unique perspective on medical imaging data by focusing on the topological features and relationships within the images at varying distance scales. This approach can reveal insights that traditional machine learning methods might overlook as it excels at identifying changes in structures (e.g., vessels, airways, organ parenchyma, etc.) [4].

Foundationally, what underlies TDA are simplicial complexes – mathematical objects that can represent complex, high-dimensional data structures [5]. In medical imaging, simplicial complexes can be used to model the intricate relationships between pixels or voxels, capturing the subtle patterns and structures that are crucial for accurate diagnosis [6].

### Advantages of TDA and simplicial complexes

1. Capturing branching and loop structures: One of the key strengths of TDA is its ability to identify and represent branching and loop structures in image data [7]. These features are particularly important in radiology, where they can indicate the presence of blood vessels, tumors, or other anatomical structures. Traditional Convolutional Neural Networks (CNNs) may struggle to capture these features due to limitations in patch size and convolution operations [8]. TDA, however, can preserve and analyze these critical structures, potentially leading to more accurate and comprehensive image interpretation.
2. N-way interactions: While many current AI models focus on pairwise interactions between pixels, TDA and simplicial complexes allow for the analysis of n-way interactions [9]. This means we can capture more complex relationships within the image data, going beyond the limitations of 2-way interactions. By considering these higher-order relationships, we can develop a more nuanced understanding of the image, potentially uncovering subtle indicators of disease or anatomical variations that might otherwise be missed [10].
3. Geometric meaning: TDA provides a way to assign geometric meaning to the relationships within medical images [11]. This geometric interpretation can improve our ability to capture and understand spatial information, which is crucial in radiology. By leveraging the geometric insights provided by TDA, we can develop more sophisticated algorithms that are better equipped to interpret the three-dimensional nature of many radiological images [12].

### Mitigating bias through TDA

The application of TDA and simplicial complexes in radiology has the potential to address and mitigate biases in several ways:

1. Improved feature extraction: By capturing more complex and subtle features of medical images, TDA can help reduce the risk of overlooking important diagnostic indicators [13]. This can be particularly valuable in cases where traditional AI models might be biased towards more common or easily detectable features.
2. Robust representation: The topological approach to data analysis is inherently more robust to certain types of noise and data variation [14]. This robustness can help reduce the impact of biases that might arise from differences in imaging equipment, patient positioning, or other external factors.
3. Interpretability: TDA offers a more interpretable framework for understanding how AI models arrive at their conclusions [15]. This increased transparency can help radiologists identify potential biases in the AI-assisted diagnostic process and make more informed decisions.
4. Diverse data representation: The ability of TDA to capture complex structures and relationships in data can help in developing more comprehensive and diverse training datasets for AI models [16]. This, in turn, can lead to algorithms that are less biased towards specific populations or anatomical variations [17].

## CONCLUSIONS

As we continue to advance the field of radiology through AI and machine learning, it is crucial that we also develop tools to identify and mitigate potential biases. TDA and the use of simplicial complexes offer a promising approach to this challenge. By providing a more comprehensive, robust, and interpretable framework for analyzing medical imaging data, TDA has the potential to enhance the accuracy and fairness of radiological assessments. While the application of TDA in radiology is still in its early stages, the potential benefits are significant. As researchers and clinicians, we must continue to explore and develop these innovative approaches to ensure that the future of AI-assisted radiology is both highly accurate and equitable for all patients.
